# Establishment and Optimization of Radiomics Algorithms for Prediction of KRAS Gene Mutation by Integration of NSCLC Gene Mutation Mutual Exclusion Information

**DOI:** 10.3389/fphar.2022.862581

**Published:** 2022-04-01

**Authors:** Jingyi Wang, Xing Lv, Weicheng Huang, Zhiyong Quan, Guiyu Li, Shuo Wu, Yirong Wang, Zhaojuan Xie, Yuhao Yan, Xiang Li, Wenhui Ma, Weidong Yang, Xin Cao, Fei Kang, Jing Wang

**Affiliations:** ^1^ Department of Nuclear Medicine, Xijing Hospital, Fourth Military Medical University, Xi’an, China; ^2^ Department of Respiratory Medicine, Xijing Hospital, Fourth Military Medical University, Xi’an, China; ^3^ School of Information Science and Technology, Northwest University, Xi’an, China

**Keywords:** non-small cell lung cancer, EGFR, KRAS, PET/CT, radiomics

## Abstract

**Purpose:** To assess the significance of mutation mutual exclusion information in the optimization of radiomics algorithms for predicting gene mutation.

**Methods:** We retrospectively analyzed 258 non-small cell lung cancer (NSCLC) patients. Patients were randomly divided into training (*n* = 180) and validation (*n* = 78) cohorts. Based on radiomics features, radiomics score (RS) models were developed for predicting KRAS proto-oncogene mutations. Furthermore, a composite model combining mixedRS and epidermal growth factor receptor (EGFR) mutation status was developed.

**Results:** Compared with CT model, the PET/CT radiomics score model exhibited higher AUC for predicting KRAS mutations (0.834 vs. 0.770). By integrating EGFR mutation information into the PET/CT RS model, the AUC, sensitivity, specificity, and accuracy for predicting KRAS mutations were all elevated in the validation cohort (0.921, 0.949, 0.872, 0.910 vs. 0.834, 0.923, 0.641, 0.782). By adding EGFR exclusive mutation information, the composite model corrected 64.3% false positive cases produced by the PET/CT RS model in the validation cohort.

**Conclusion:** Integrating EGFR mutation status has potential utility for the optimization of radiomics models for prediction of KRAS gene mutations. This method may be used when repeated biopsies would carry unacceptable risks for the patient.

## Introduction

Lung cancer is the leading cause of cancer death globally in 2022 (Sung et al., 2021; Siegel et al., 2022). Non-small cell lung cancer (NSCLC) accounts for about 85% of total lung cancer cases (Molina et al., 2008). Tyrosine kinase inhibitors (TKI) have clinical utility as the standard first-line therapy drugs for NSCLC patients with mutations in genes encoding epidermal growth factor receptor (EGFR), anaplastic lymphoma kinase (ALK) or ROS proto-oncogene 1 (ROS1) (Drusbosky et al., 2021; Nagasaka et al., 2021; Popat et al., 2021). Therefore, determination of genetic status is a prerequisite for targeting therapy and avoiding treatments with little clinical benefit (NCCN, 2022).

In NSCLC, over 80% of total gene mutations are accounted for by EGFR (50%) and KRAS proto-oncogene (30%) (Hirsch and Bunn, 2009; Skoulidis et al., 2021). EGFR-targeted TKIs (EGFR-TKI), such as gefitinib, afatinib and osimertinib have been widely used in the clinic. More recently, KRAS mutation-targeting drugs, such as sotorasib (AMG510) and Adagrasib (MRTX849), have been shown to elicit a 37.1–45% overall response rate in clinical trials (Palma et al., 2021). International guidelines have recommended testing for the conventional mutations, EGFR, B-Raf, ALK and ROS1(Kerr et al., 2021), in advanced NSCLC with KRAS only being included in June 2021 (NCCN NSCLC guidelines). Current ASCO, ESMO and CSCO guidelines do not include KRAS in the list of testing recommendations. Whereas some patients may have been tested for KRAS mutations to predict responsiveness to EGFR-TKI therapy, many will only receive mutation testing according to ESMO and ASCO guidelines. For the latter group, knowledge of the KRAS mutation status is particularly important to inform the choice of newly developed KRAS mutation-targeting agents. Repeated biopsies are invasive and next-generation sequencing (NGS) is expensive (Santos et al., 2019; Kerr et al., 2021; Mantilla et al., 2021). The non-invasive approach of radiomics may provide a solution to prediction of KRAS mutation status.

With recent advances in artificial intelligence, radiomics has been widely used in the prediction of gene mutations (Mu et al., 2020; Zhang et al., 2020), and the performance of these radiomics models has been subject to continuous optimization (Wu et al., 2021). To improve the accuracy in predicting gene mutations, previous studies integrated various clinical information, including smoking history, radiographic features, and serum tumor markers into radiomics models (Zhang et al., 2020; Chang et al., 2021; Ren et al., 2021; Weng et al., 2021). However, because of the weak theoretical connection between the above clinical information and genetic mutations, a better optimization method may be potentially achieved by integrating more directly relevant genetic information.

Previous work has shown the mutual exclusivity of multiple gene mutations, including EGFR and KRAS, a phenomenon referred to as gene mutation mutual exclusion (Shigematsu and Gazdar, 2006; Kim et al., 2015; Mok et al., 2016). Therefore, in theory, EGFR mutation status should be related to KRAS mutation status, an observation which could be exploited in optimizing the accuracy of KRAS radiomics models. The current study aims to verify whether knowledge of EGFR mutation status could be used to improve the accuracy of the radiomics model for predicting KRAS mutation status based on ^18^F-FDG PET/CT multimodality imaging data.

## Materials and Methods

### Patients

We retrospectively analyzed the PET/CT images of NSCLC patients diagnosed pathologically in Xijing Hospital from 2016 to 2020. A total of 258 NSCLC patients were screened, all of which received EGFR and KRAS genetic testing at the primary site of lung cancer. It should be noted that since KRAS is not a conventional recommended target for testing in ESMO, CAP/IASLC/AMP, and Pan-Asian guidelines, patients undergoing additional sequencing are rare in current clinical practice (Kerr et al., 2021). Inclusion criteria were: 1) confirmation of NSCLC by pathology or cytology; 2) having undergone ^18^F-FDG PET/CT imaging; 3) primary lesion diameter > 1cm; 4) no history of other malignant tumors. Exclusion criteria were: 1) having received radiotherapy or chemotherapy before PET/CT examination; 2) poor PET/CT image quality. This retrospective study was approved by the Medical Ethics Committee of Xijing Hospital (Approval No. KY20173008-1).

All cases were randomly assigned in a 7:3 ratio to the training cohort (n = 180) or validation cohort (*n* = 78). All cases in the training cohort were used to train the predictive model, while cases in the validation cohort were used to independently evaluate the model’s performance.

### ^18^F-FDG PET/CT Imaging

All patients received ^18^F-FDG PET/CT scans on the same equipment (Biograph 40, Siemens), following a standard clinical protocol (Delbeke et al., 2006). Briefly, patients were required to fast for >6 h before the scan and exhibit blood glucose control within 7.8 mmol/L. CT parameters were 100 kV, 110 mAs, 0.5 s rotation time, 3 mm slice thickness, 700 mm field of view, and 512 × 512 matrix. For PET scanning, 4.44–5.55 MBq/kg of ^18^F-FDG was injected. Scanning was initiated 60 min after tracer administration, with 3 min scans per bed position. PET and CT images were reconstructed using an ordered-subsets expectation-maximization algorithm with four iterations and eight subsets (Kang et al., 2016; Kang et al., 2019; Kang et al., 2020).

### Tumor Segmentation, Feature Extraction, and Selection

All regions of interest (ROI) were defined for PET/CT images by two experienced nuclear medicine physicians using MITK (Medical Imaging Interaction Toolkit v2018.04.2) software, as described previously (Chang et al., 2021; Han et al., 2021; Wu et al., 2021; Zhou H. et al., 2021). ROIs identified by CT were manually outlined slice-by-slice by nuclear medicine physicians in the lung window (WW: 1500HU, WL: −500HU). ROIs identified from PET images were segmented by semi-automatic outlining using the “region growing 3D tools” in MITK software, referencing the 3D-ROI with a standard uptake value (SUV) threshold of 40%. For lesion boundaries close to the heart or chest wall, PET image ROIs were outlined manually with reference to the CT image.

SUVs of PET images were converted prior to feature extraction. Radiomics features (including first order features, texture features and shape features) were extracted using the Pyradiomics software package (Koyasu et al., 2020; Yu et al., 2021; Zhang et al., 2021; Zhou Y. et al., 2021). Nine image filters (wavelet, lbp2D, lbp3D, Laplacian of Gaussian, square root, square, gradient, logarithm, exponential) were used to analyze high-dimensional image features. Sigma parameters were 1.0, 2.0, 3.0, 4.0, 5.0 when using the Laplacian of Gaussian filter. A bin width of 25 (CT) and 0.1 (PET) voxel and an array shift of 1000 (CT) and 0 (PET) were used for feature extraction. All data were subjected to standardized data preprocessing.

Feature selection was performed using univariate and multivariate analyses with a stepwise selection method. To avoid overfitting of the model, Spearman analysis was used to determine the correlation between radiomics features and KRAS gene mutations. The threshold for Spearman correlation analysis was 0.3. Mann–Whitney U tests were performed to identify features with a statistical threshold of *p* < 0.05. Least absolute shrinkage and selection operator (LASSO) was used to select optimal features (Zhang et al., 2020).

### Model Establishment, Comparison, and Evaluation

CT radiomics score (RS) and mixed PET/CT RS (mixedRS) were calculated for each patient based on the optimal feature subsets screened by LASSO. A CT RS model and PET/CT RS model were built by logistic regression. RS was then optimized by incorporating EGFR mutation information to develop the composite model by logistic regression. A nomogram was constructed based on the composite model. Calibration curves were plotted to evaluate the goodness of fit of the prediction model and the three models were compared in training and validation cohorts. Performance parameters included the AUC, accuracy, sensitivity, specificity, false positive rate (FPR), false negative rate (FNR) and Youden index (YI). AUCs of the three models were compared by Delong test. Decision curve analysis was performed to compare the clinical benefit of the three models and data balanced using the synthetic minority oversampling technique (SMOTE). SMOTE is a powerful over-sampling method that has shown a great deal of successes in class imbalanced problems (Fotouhi et al., 2019). The minority class is over-sampled by taking each sample and introducing synthetic examples along the line segments joining any/all of the nearest neighbors of the k minority class (Chawla et al., 2002).

### Statistical Analysis

All statistical analyses were performed using R software version 3.5.1 and Python software version 3.5.6. T-tests and Mann–Whitney U tests were used to compare continuous variables, while Chi-squared tests were used to compare differences in categorical variables. A *p*-value <0.05 indicated statistical significance.

## Results

### Patient Characteristics

Clinical characteristics of the 258 patients in training and validation cohorts are shown in Table 1. Chi-square testing revealed that EGFR mutation was a significant predictor of KRAS mutation in both the training and the validation cohorts (both *p* < 0.001).

**TABLE 1:** Characteristics of patients in predicting KRAS mutations.

Characteristic	Training cohort (*n* = 180)		Validation cohort (*n* = 78)	
	KRAS wild type (*n* = 90)	KRAS mutant (*n* = 90)	KRAS wild type (*n* = 39)	KRAS mutant (*n* = 39)
Age, years (mean ± SD)	61.13 ± 11.26	64.76 ± 10.48	59.33 ± 10.76	64.18 ± 11.15
Gender				
Male	61 (67.78%)	67 (66.67%)	21 (53.85%)	31 (79.49%)
Female	29 (32.22%)	23 (25.56%)	18 (46.15%)	8 (20.51%)
Smoking				
Yes	52 (57.78%)	64 (71.11%)	20 (51.28%)	30 (76.92%)
No	38 (42.22%)	26 (28.89%)	19 (48.72%)	9 (23.08%)
CEA (ng/ml)	5.97 (3.20, 20.44)	4.57 (2.99, 6.52)	5.13 (2,65, 18.28)	4.60 (3.14, 10.35)
EGFR				
Wild type	46 (51.11%)	90 (100.00%)	21 (53.85%)	39 (100.00%)
Mutant	44 (48.89%)	0 (0.00%)	18 (46.15%)	0 (0.00%)
SUVmax	9.85 (6.70, 13.07)	7.79 (5.14, 11.54)	9.48 (7.65, 13.79)	10.37 (6.85, 12.42)
MTV	30.50 (17.34, 98.34)	31.95 (19.02, 100.55)	24.11 (13.81, 64.65)	27.56 (18.94, 138.08)

Note: CEA, carcinoembryonic antigen; SUV, standard uptake value; MTV, metabolic tumor volume.

### Feature Selection and RS Establishment

**FIGURE 1:**
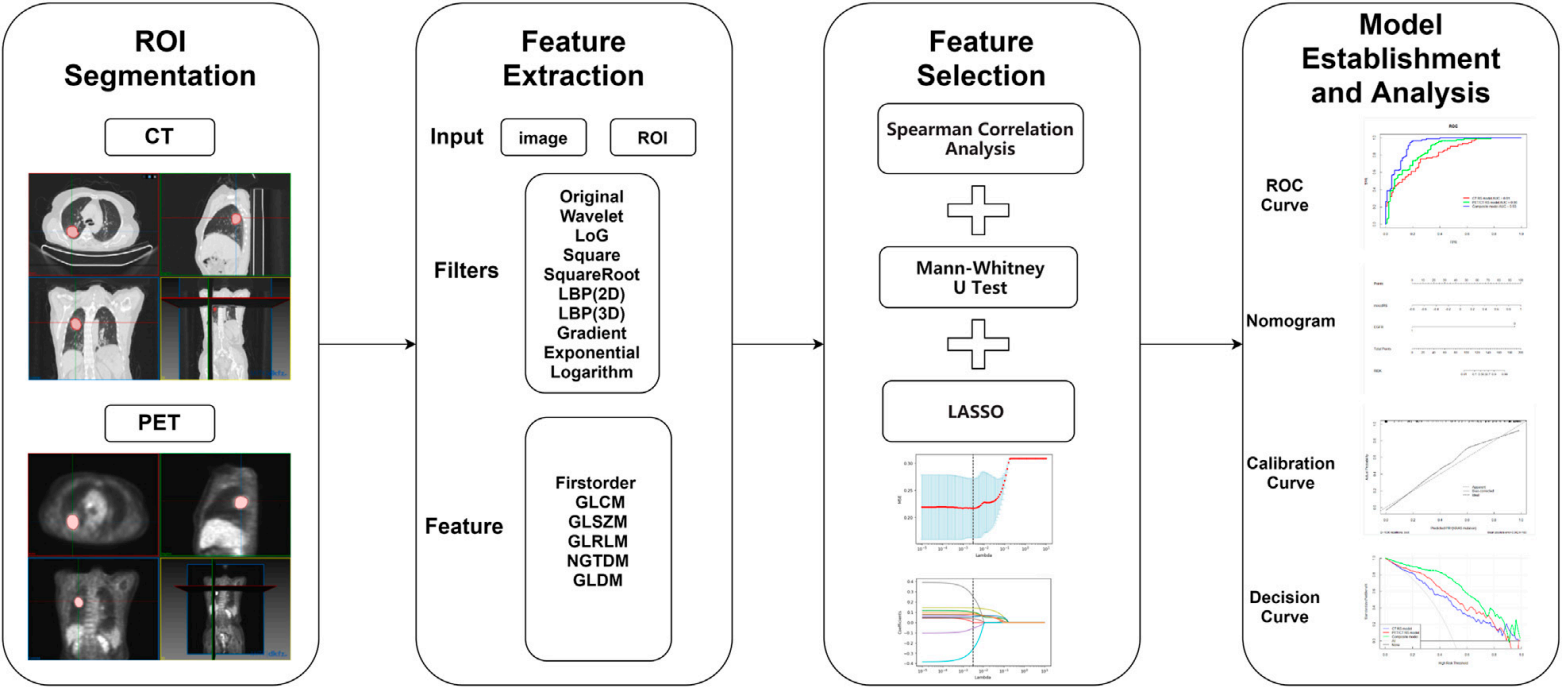
Study workflow.

**FIGURE 2:**
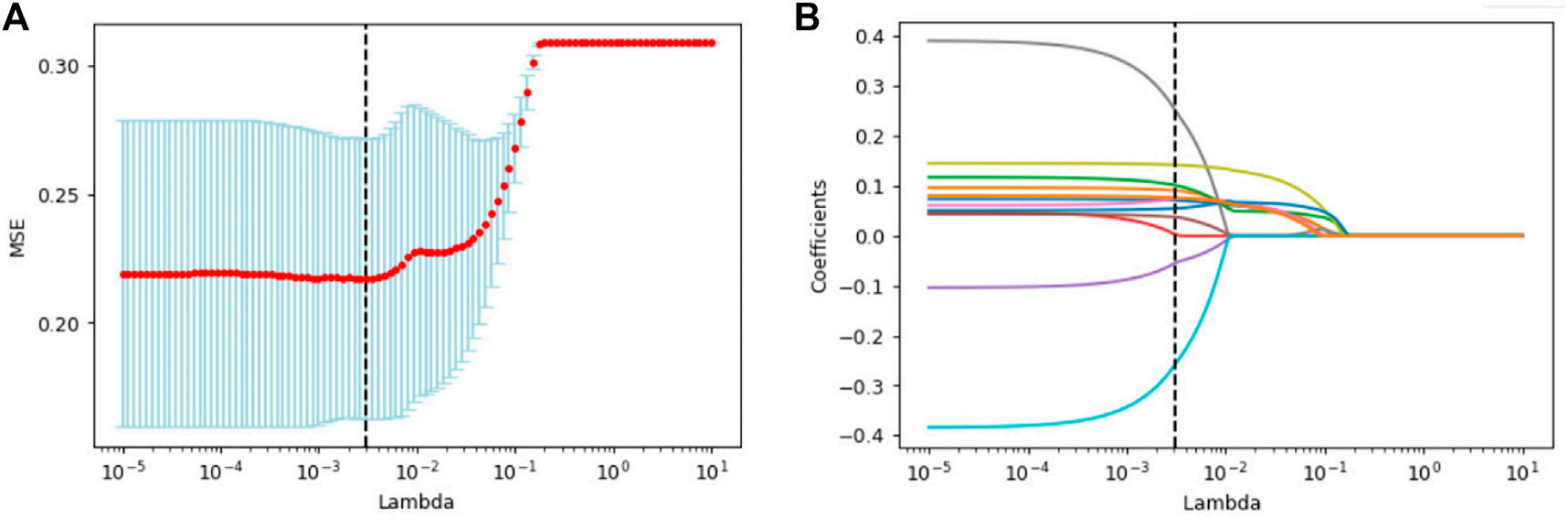
The LASSO and 10-fold cross-validation were used to select the optimal radiomics features. 12 features corresponding to the optimal lambda values were selected. **(A)** Mean square error path. **(B)** LASSO coefficient profiles of radiomics features.

The scheme for establishment of the radiomics model is presented in Figure 1. A total of 4306 features were extracted in accordance with the image biomarker standardization initiative (IBSI) (Zwanenburg et al., 2017). After univariate analysis and LASSO screening, four features were included in the CT RS model and 12 features in the PET/CT RS model (4 CT plus 8 PET features; Figure 2; Supplementary Material). The RS was calculated using the Supplementary Formulae of RS. The CT RS and mixedRS was significantly different between patients with KRAS mutations and those with wild type KRAS in both the training and the validation cohorts (both *p* < 0.001). See Supplementary Table S1 for detailed information.

### Comparison of the Three Models and the Establishment of the Nomogram

**FIGURE 3:**
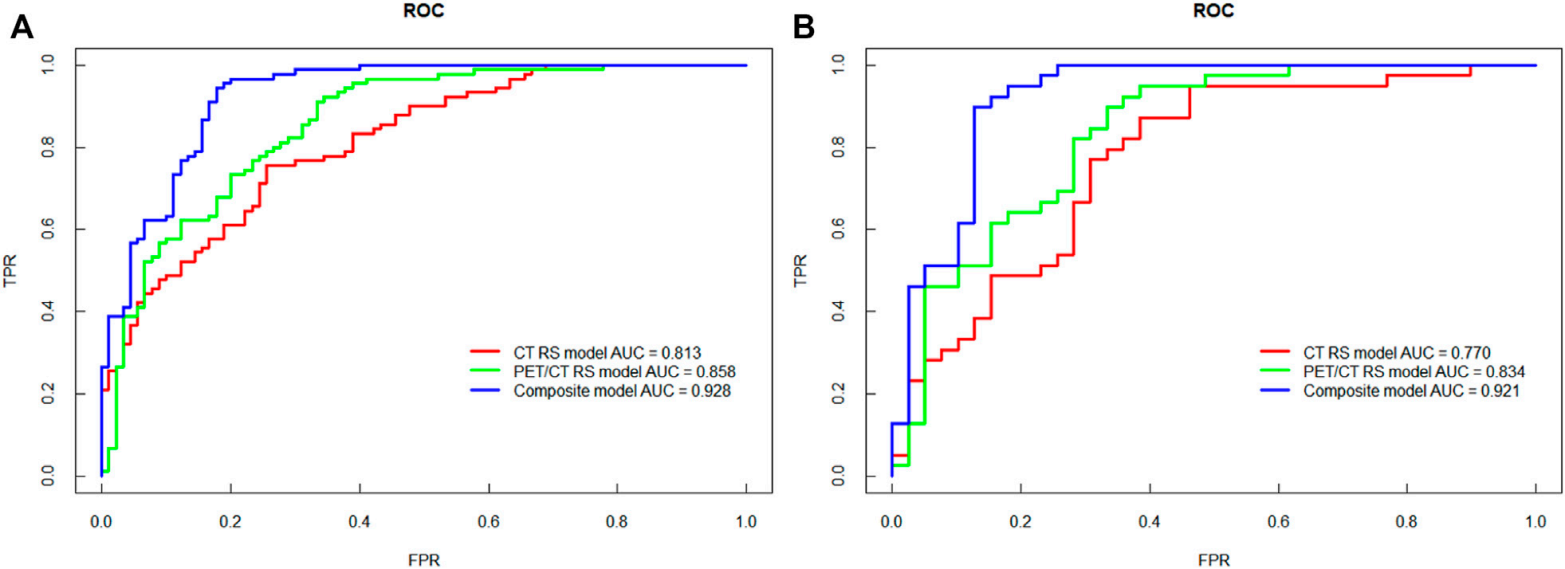
ROC curves of the three models for predicting KRAS mutations. **(A)** The ROC curve of the training cohort. **(B)** The ROC curve of the validation cohort.

In the validation cohort, the AUC, sensitivity, specificity, accuracy and YI of the PET/CT RS model were superior to the CT RS model (AUC: 0.834 vs. 0.770, sensitivity: 0.923 vs. 0.872, specificity: 0.641 vs. 0.615, accuracy: 0.782 vs. 0.744, YI: 0.564 vs. 0.487; Figure 3). Therefore, we established composite model combining mixedRS and EGFR mutation information. The composite model performed better than the PET/CT RS model in both cohorts of the study, with an AUC of 0.928 (95% CI [0.890, 0.965]) in the training cohort and 0.921 (95% CI [0.856, 0.986]) in the validation cohort. The Delong test revealed a significant difference between the AUC of the PET/CT RS model and the composite model in both the training and the validation cohorts (*p* < 0.001 and *p* = 0.012, respectively). Data used for comparison of the three models are shown in Table 2. A PET RS model was constructed in a similar manner (data shown in Table 2) for comparative purposes, although PET examination alone is rarely used in clinical practice.

**TABLE 2:** Diagnostic performance of models for predicting KRAS mutations.

Model	Training cohort							Validation cohort						
	AUC (95%CI)	Sen	Spe	Acc	FPR(%)	FNR (%)	YI	AUC (95%CI)	Sen	Spe	Acc	FPR(%)	FNR (%)	YI
CT RS model	0.813 (0.752, 0.873)	0.756	0.744	0.756	25.6	24.4	0.500	0.770 (0.664, 0.876)	0.872	0.615	0.744	38.5	12.8	0.487
PET RS model	0.840 (0.748, 0.932)	0.833	0.767	0.800	23.3	20.0	0.600	0.777 (0.705, 0.849)	0.615	0.897	0.756	10.3	38.5	0.512
PET/CT RS model	0.858 (0.804, 0.912)	0.922	0.656	0.789	34.4	7.8	0.578	0.834 (0.742, 0.925)	0.923	0.641	0.782	35.9	7.7	0.564
Composite model	0.928 (0.890, 0.965)	0.956	0.811	0.883	18.9	4.4	0.767	0.921 (0.856, 0.986)	0.949	0.872	0.910	12.8	5.1	0.821

Note: Sen: Sensitivity; Spe: Specificity; Acc: Accuracy; FPR: false positive rate; FNR: false negative rate; YI: Youden index; CI: confidence interval.

**FIGURE 4:**
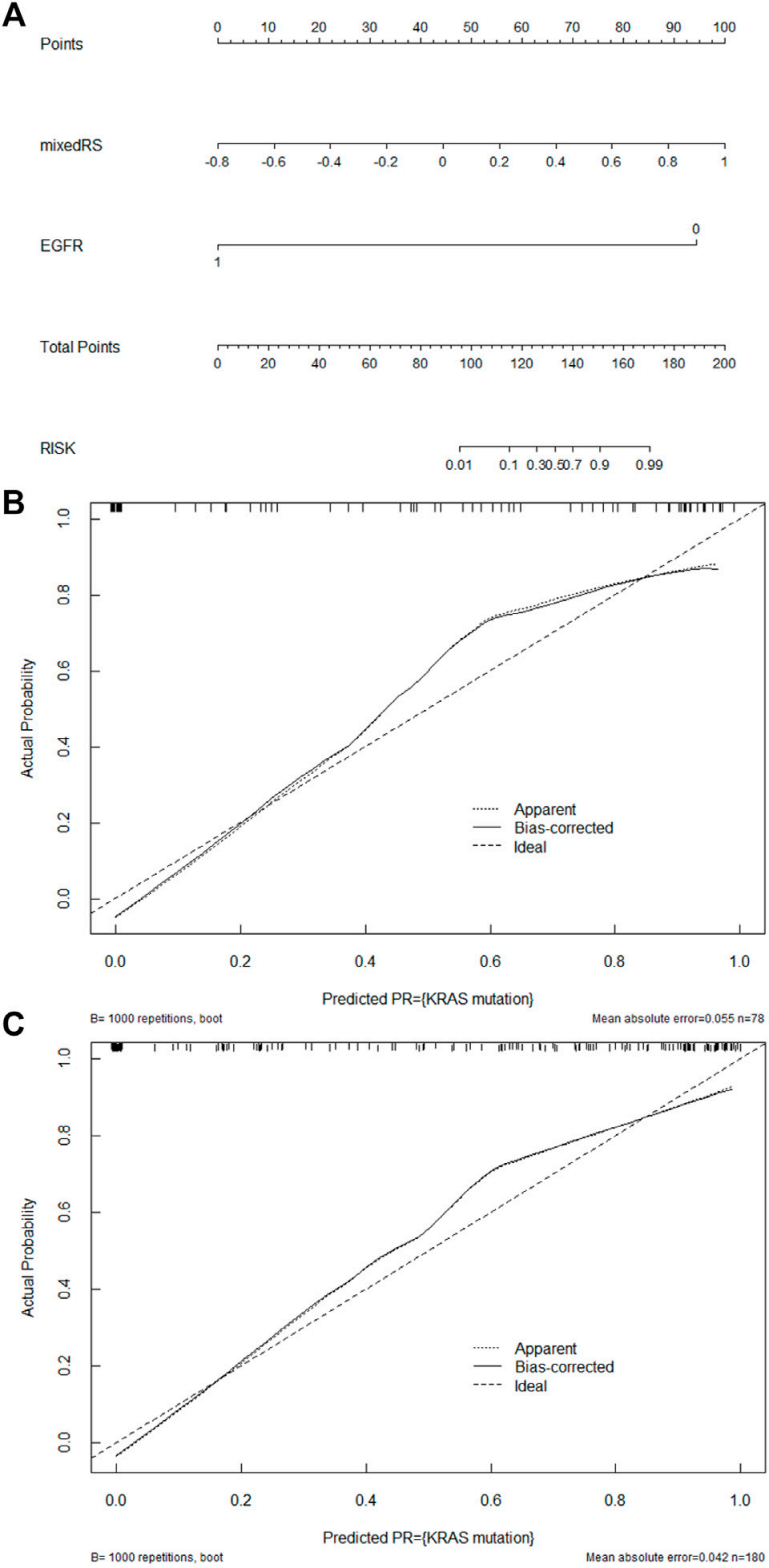
Development and performance of a nomogram. **(A)** Nomogram establishment by integrating mixedRS and EGFR. Nomogram calibration curves in the training **(B)** and validation **(C)** cohorts. The diagonal dashed line represents a predicted value equal to the true value, and the solid line is the model’s prediction of KRAS mutation. The closer the two lines are, the better the performance.

The FPR of the composite model (12.8%) was significantly decreased compared to the PET/CT RS model (35.9%) in validation cohort, and no additional false negative error was generated. In the validation cohort, the composite model corrected 64.3% false positive errors generated by the PET/CT RS model. The nomogram that integrates mixedRS and EGFR was created for further clinical use in predicting KRAS mutation status (Figure 4A). The nomogram calibration curves are presented in Figures 4B,C, which indicated good consistency between the predicted and actual values.

### Decision Curve Analysis

**FIGURE 5:**
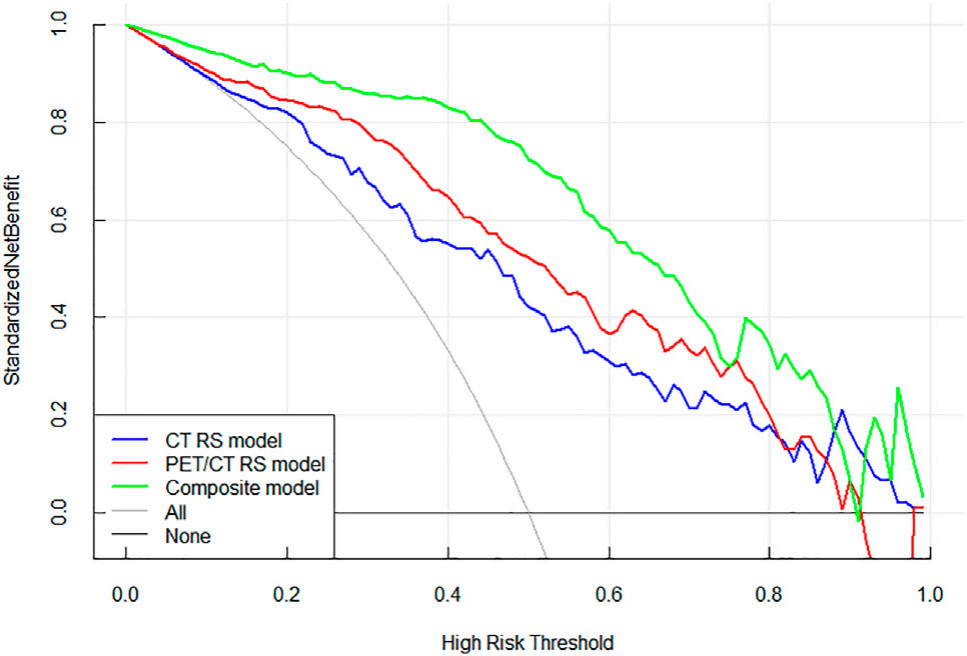
Decision curves of the three models. The green line represents the composite model incorporating mixedRS and EGFR. The blue and red lines represent CT RS model and PET/CT RS model, respectively. The grey line indicates the assumption that all patients possess the gene mutation, while the black line indicates the assumption that all patients possess the wild type gene.

To evaluate the clinical usefulness of the established radiomics models, decision curves were drawn (Figure 5). The composite model had higher net benefit than CT RS model and PET/CT RS model when the threshold probability reached <90%.

## Discussion

Two findings of the current study may be highlighted. Firstly, three radiomics prediction models for KRAS mutations were established: CT RS model, PET/CT RS model and composite model combining mixedRS and EGFR. Addition of PET information improved the accuracy of KRAS prediction. Secondly, EGFR information was found to be of benefit in establishing a radiomics model to predict KRAS mutation status, a finding that has clinical significance for patients who would otherwise have to suffer repeated biopsies. Relative to the PET/CT RS model, the composite mixedRS and EGFR gene status model showed improved accuracy and AUC for predicting KRAS mutations without a significant effect on sensitivity.

Analysis of the RS formulae developed during the current study revealed a link between radiomics features and gene mutations. CT_wavelet-HHL_firstorder_Skewness, associated with the asymmetry of value distribution about the mean, and CT_wavelet-LHH_gldm_DNUN, associated with the similarity of dependence throughout the image, were both positively correlated with KRAS mutations in both the CT RS model and the PET/CT RS model. Furthermore, PET_lbp-2D_gldm_GLV, associated with the variance in grey level, and PET_lbp-2D_firstorder_Variance, associated with the squared distances of each intensity value from the mean value, were both negatively correlated with KRAS mutations. Interpretation of the features of radiomics remains difficult which limits the clinical applications. More research is required to verify the theoretical link between radiomic features and clinical outcome.

Radiomics has been widely used in the prediction of genetic mutations since 2012 with much effort being expended in optimization of the approach. Zhang et al. combined clinical information, such as smoking and gender, with a radiomics model to improve the prediction of EGFR mutations in lung cancer compared with a radiomics model alone (Zhang et al., 2020). Although non-smoking women have been shown to be more likely to have EGFR mutations (Lynch et al., 2004; Dogan et al., 2012), there is still no direct correlation between this clinical information and gene mutation status.

Expert consensus, represented by the latest NCCN guidelines, affirms the existence of the mutually exclusive phenomenon among genetic mutations, especially between EGFR and KRAS genes (Marchetti et al., 2005; Amado et al., 2008; Roosan et al., 2021; NCCN, 2022). Although the mechanism is not fully understood, it may be that tumors with KRAS mutations have already activated further downstream effectors and removing the requirement for EGFR mutations (NCCN, 2022). Therefore, the mutually exclusive genetic phenomenon is more directly correlated to gene mutation status than other clinical information. The current study confirms that clinical information regarding EGFR can be used to optimize radiomics algorithms for prediction of KRAS gene mutations.

Hitherto, most patients have only been tested for mutations in a limited number of genes, such as EGFR. With the advent of KRAS-targeted drugs, including sotorasib, and RET-targeted, pralsetinib (Thein et al., 2021), version 5.2021 of the NCCN guidelines for NSCLC has been amended to indicate the need for KRAS genetic testing. Puncture biopsy is risky with potential outcomes such as pneumothorax, hemoptysis and even death. However, the current findings demonstrate a non-invasive method to predict KRAS status based on pre-existing results of mutation testing. This method not only has the potential to avoid secondary biopsies for patients at risk but also to guide patient selection for new KRAS-targeted drugs.

We acknowledge some limitations to the current study. Firstly, as a single center retrospective study, few KRAS mutation cases were included since KRAS was not routinely tested. This limitation could be overcome by further validation in multi-center studies with expanded sample sizes. Secondly, only EGFR and KRAS mutations were investigated with other mutations, such as ALK and ROS1, not included. The prevalence of ALK and ROS1 mutations in NSCLC patients is 2–7% and 1–2%, respectively, with the mutations only occurring in 1.2% of patients enrolled in the current study. More research is needed to confirm the value of ALK and ROS1 mutations in optimizing the radiomics model. Thirdly, the current results were obtained from imaging data and samples of primary lesions. Tumors are highly heterogeneous and mutation status may differ between the primary and metastatic lesions or even within a single lesion. Further studies are warranted to address more complex issues related to the heterogeneity of mutations. In conclusion, although radiomics features are difficult to interpret, the current approach may have utility as a complementary method in the clinic, particularly among patients for whom repeated biopsies carry unacceptable risks.

## Data Availability

The original contributions presented in the study are included in the article/Supplementary Material, further inquiries can be directed to the corresponding authors.
