# Supplementary material for: Establishment and Optimization of Radiomics Algorithms for Prediction of KRAS Gene Mutation by Integration of NSCLC Gene Mutation Mutual Exclusion Information
Source: Front Pharmacol. 2022 Apr 1;13:862581. doi: 10.3389/fphar.2022.862581 (PMC9010886; doi:10.3389/fphar.2022.862581)
Supplement: Supplementary file 1 [file DataSheet1.DOCX]

Supplementary Material

**Supplementary material of 12 selected radiomics features**

CT_square_glszm_SizeZoneNonUniformityNormalized (CT_square_glszm_SZNUN)

CT_wavelet-LHH_gldm_DependenceNonUniformityNormalized (CT_wavelet-LHH_gldm_DNUN)

CT_wavelet-HHL_firstorder_Skewness

CT_wavelet-HHL_gldm_DependenceNonUniformityNormalized (CT_wavelet-HHL_gldm_DNUN)

PET_lbp-2D_glrlm_ShortRunLowGrayLevelEmphasis (PET_lbp-2D_glrlm_SRLGLE)

PET_lbp-2D_glrlm_ShortRunEmphasis (PET_lbp-2D_glrlm_SRE)

PET_lbp-2D_gldm_GrayLevelVariance (PET_lbp-2D_gldm_GLV)

PET_original_glcm_MaximumProbability (PET_original_glcm_MP)

PET_lbp-2D_gldm_SmallDependenceLowGrayLevelEmphasis (PET_lbp-2D_gldm_SDLGLE)

PET_lbp-2D_glcm_DifferenceVariance (PET_lbp-2D_glcm_DV)

PET_lbp-2D_firstorder_Variance, PET_gradient_firstorder_Kurtosis

**Supplementary Formulae of RS**

CT RS = CT_wavelet-HHL_firstorder_Skewness×0.145249+CT_square_glszm_SZNUN×0.086535+CT_wavelet-HHL_gldm_DNUN×0.103472+CT_wavelet-LHH_gldm_DNUN×0.079215+0.375281

mixedRS = CT_square_glszm_SZNUN×0.071103+CT_wavelet-LHH_gldm_DNUN×0.091237+PET_lbp-2D_glrlm_SRLGLE×0.101662+PET_lbp-2D_glrlm_SRE×0.003222+PET_lbp-2D_gldm_GLV× (-0.003286) +PET_original_glcm_MP×0.037816+PET_lbp-2D_gldm_SDLGLE×0.074111+PET_lbp-2D_glcm_DV×0.253524+CT_wavelet-HHL_firstorder_Skewness×0.141952+PET_lbp-2D_firstorder_Variance× (-0.311782) +CT_wavelet-HHL_gldm_DNUN×0.054750+PET_gradient_firstorder_Kurtosis×0.076734+0.490000

**Supplementary Table S1.** RS in predicting KRAS mutations.

|  | Training cohort  (n=180) | | | Validation cohort  (n=78) | | |
| --- | --- | --- | --- | --- | --- | --- |
| RS | **KRAS wild type**  **(n=90)** | **KRAS mutant**  **(n=90)** | ***P*** | **KRAS wild type**  **(n=39)** | **KRAS mutant**  **(n=39)** | ***P*** |
| CT RS  Median  (first quartile, third quartile) | -0.173  (-0.266, -0.036) | 0.132  (-0.044, 0.319) | <0.001 | -0.170  (-0.237, 0.060) | 0.079  (-0.082, 0.313) | <0.001 |
| mixedRS  Median  (first quartile, third quartile) | -0.240  (-0.333, -0.031) | 0.191  (-0.016, 0.372) | <0.001 | -0.223  (-0.320, -0.001) | 0.205  (-0.040, 0.318) | <0.001 |

**Supplementary Table S2** List of Extracted Radiomics Features.

| **Classification (Total number=2153)** | **Features** |
| --- | --- |
| **First Order Features (18)** | Energy TotalEnergy Entropy Minimum  10th percentile 90th percentile Maximum Mean  Median Interquartile Range Range Skewness  Kurtosis Variance Uniformity  Mean Absolute Deviation (MAD) Robust Mean Absolute Deviation (rMAD)  Root Mean Squared (RMS) |
| **Shape Features (14)** | Elongation Flatness LeastAxisLength MajorAxisLength  Maximum2DDiameterColumn Maximum2DDiameterRow VoxelVolume  Maximum2DDiameterSlice Maximum3DDiameter shape_MeshVolume  MinorAxisLength Sphericity SurfaceArea SurfaceVolumeRatio |
| **Textural Features (24+16+16+14+5=75)** | **24 features from GLCM:**  Autocorrelation Joint Average Cluster Prominence Cluster Shade  Cluster Tendency Contrast Correlation Difference Average  Difference Entropy Difference Variance Joint Energy Joint Entropy  Informational Measure of Correlation (IMC) 1  Informational Measure of Correlation (IMC) 2  Inverse Difference Moment (IDM)  Maximal Correlation Coefficient (MCC)  Inverse Difference Moment Normalized (IDMN)  Inverse Difference (ID)   Inverse Difference Normalized (IDN)  Inverse Variance (IV) Maximum Probability Sum Average  Sum Entropy Sum of Squares  **16 features from GLRLM:**  Short Run Emphasis (SRE)  Long Run Emphasis (LRE)  Gray Level Non-Uniformity (GLN)  Gray Level Non-Uniformity Normalized (GLNN)  Run Length Non-Uniformity (RLN)  Run Length Non-Uniformity Normalized (RLNN)  Run Percentage (RP)  Gray Level Variance (GLV)  Run Variance (RV)  Run Entropy (RE)  Low Gray Level Run Emphasis (LGLRE)  High Gray Level Run Emphasis (HGLRE)  Short Run Low Gray Level Emphasis (SRLGLE)  Short Run High Gray Level Emphasis (SRHGLE)  Long Run Low Gray Level Emphasis (LRLGLE)  Long Run High Gray Level Emphasis (LRHGLE)  **16 features from GLSZM:**  Small Area Emphasis (SAE)  Large Area Emphasis (LAE)  Gray Level Non-Uniformity (GLN)  Gray Level Non-Uniformity Normalized (GLNN)  Size-Zone Non-Uniformity (SZN)  Size-Zone Non-Uniformity Normalized (SZNN)  Zone Percentage (ZP)  Gray Level Variance (GLV)  Zone Variance (ZV)  Zone Entropy (ZE)  Low Gray Level Zone Emphasis (LGLZE)  High Gray Level Zone Emphasis (HGLZE)  Small Area Low Gray Level Emphasis (SALGLE)  Small Area High Gray Level Emphasis (SAHGLE)  Large Area Low Gray Level Emphasis (LALGLE)  Large Area High Gray Level Emphasis (LAHGLE)  **14 features from GLDM:**  Small Dependence Emphasis (SDE)  Large Dependence Emphasis (LDE)  Gray Level Non-Uniformity (GLN)  Dependence Non-Uniformity (DN)  Dependence Non-Uniformity Normalized (DNN)  Gray Level Variance (GLV)  Dependence Variance (DV)  Dependence Entropy (DE)  Low Gray Level Emphasis (LGLE)  High Gray Level Emphasis (HGLE)  Small Dependence Low Gray Level Emphasis (SDLGLE)  Small Dependence High Gray Level Emphasis (SDHGLE)  Large Dependence Low Gray Level Emphasis (LDLGLE)  Large Dependence High Gray Level Emphasis (LDHGLE)  **5 features from NGTDM:**  Coarseness Contrast  Busyness Complexity  Strength |
| **Wavelet features (93*8=744)** | **8 decompositions:**  For each decomposition, all of the above features except shape features were computed. |
| **LoG features (93*5)** | **5 decompositions:**  For each decomposition, all of the above features except shape features were computed. |
| **Square features (93)** | **1 decompositions:**  For each decomposition, all of the above features except shape features were computed. |
| **SquareRoot features (93)** | **1 decompositions:**  For each decomposition, all of the above features except shape features were computed. |
| **Local Binary Pattern (2D) features (93)** | **1 decompositions:**  For each decomposition, all of the above features except shape features were computed. |
| **Local Binary Pattern (3D) features (93*3=279)** | **3 decompositions:**  For each decomposition, all of the above features except shape features were computed. |
| **Gradient features (93)** | **1 decompositions:**  For each decomposition, all of the above features except shape features were computed. |
| **Exponential features (93)** | **1 decompositions:**  For each decomposition, all of the above features except shape features were computed. |
| **Logarithm features (93)** | **1 decompositions:**  For each decomposition, all of the above features except shape features were computed. |
